# Supplementary figures and images for: Genome-wide DNA polymorphisms in four Actinidia arguta genotypes based on whole-genome re-sequencing
Source: PLoS One. 2020 Apr 10;15(4):e0219884. doi: 10.1371/journal.pone.0219884 (PMC7147731; doi:10.1371/journal.pone.0219884)

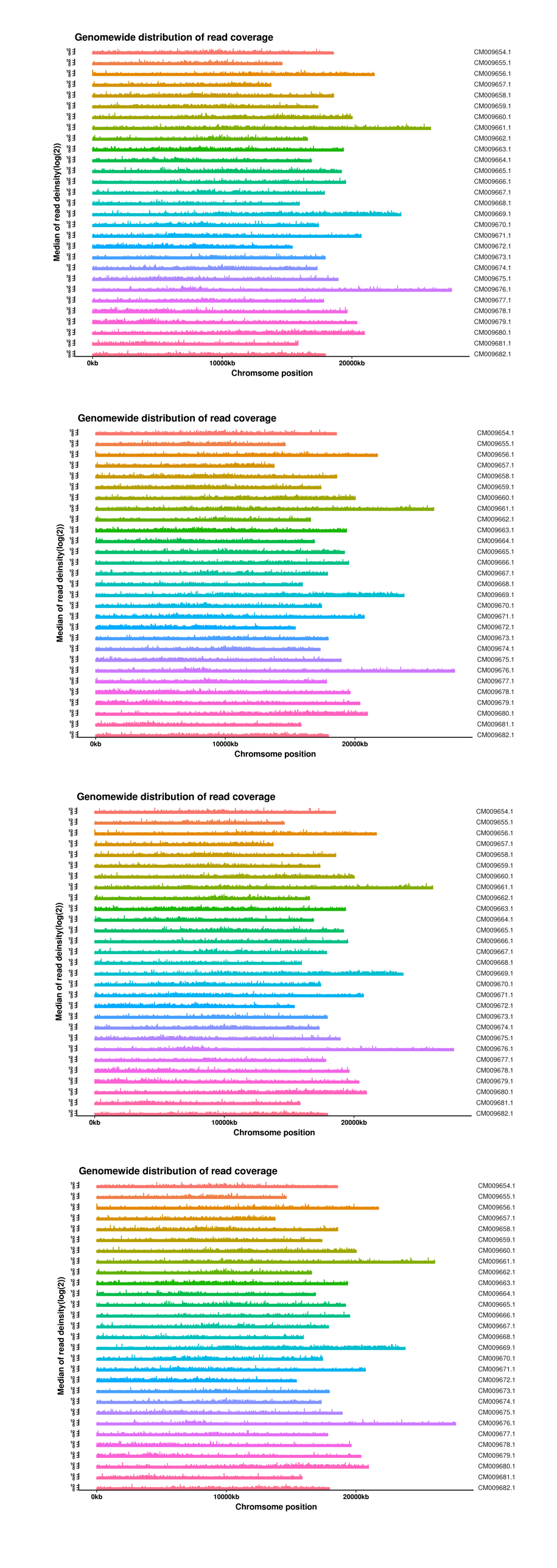

Supplement: S1 Fig — A: ‘Ruby-3’, B: ‘Hongbei male’, C: ‘Yongfeng male’, D: ‘Kuilv male’. (TIF) [file pone.0219884.s001.tif]
