# Supplementary material for: Genome-wide DNA polymorphisms in four Actinidia arguta genotypes based on whole-genome re-sequencing
Source: PLoS One. 2020 Apr 10;15(4):e0219884. doi: 10.1371/journal.pone.0219884 (PMC7147731; doi:10.1371/journal.pone.0219884)
Supplement: S2 Fig — (PDF) [file pone.0219884.s002.pdf]

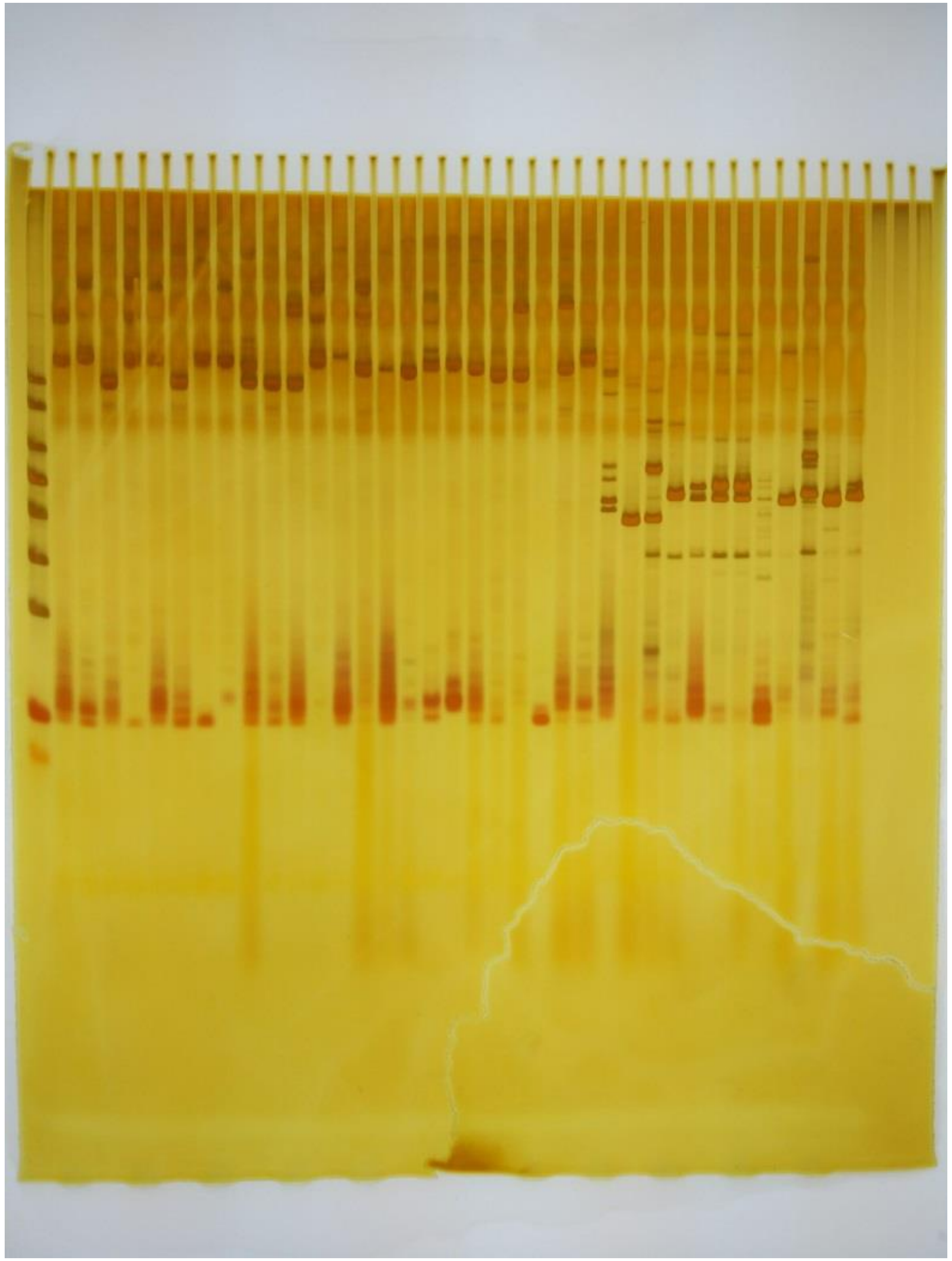

Chr3 lane2-lane12

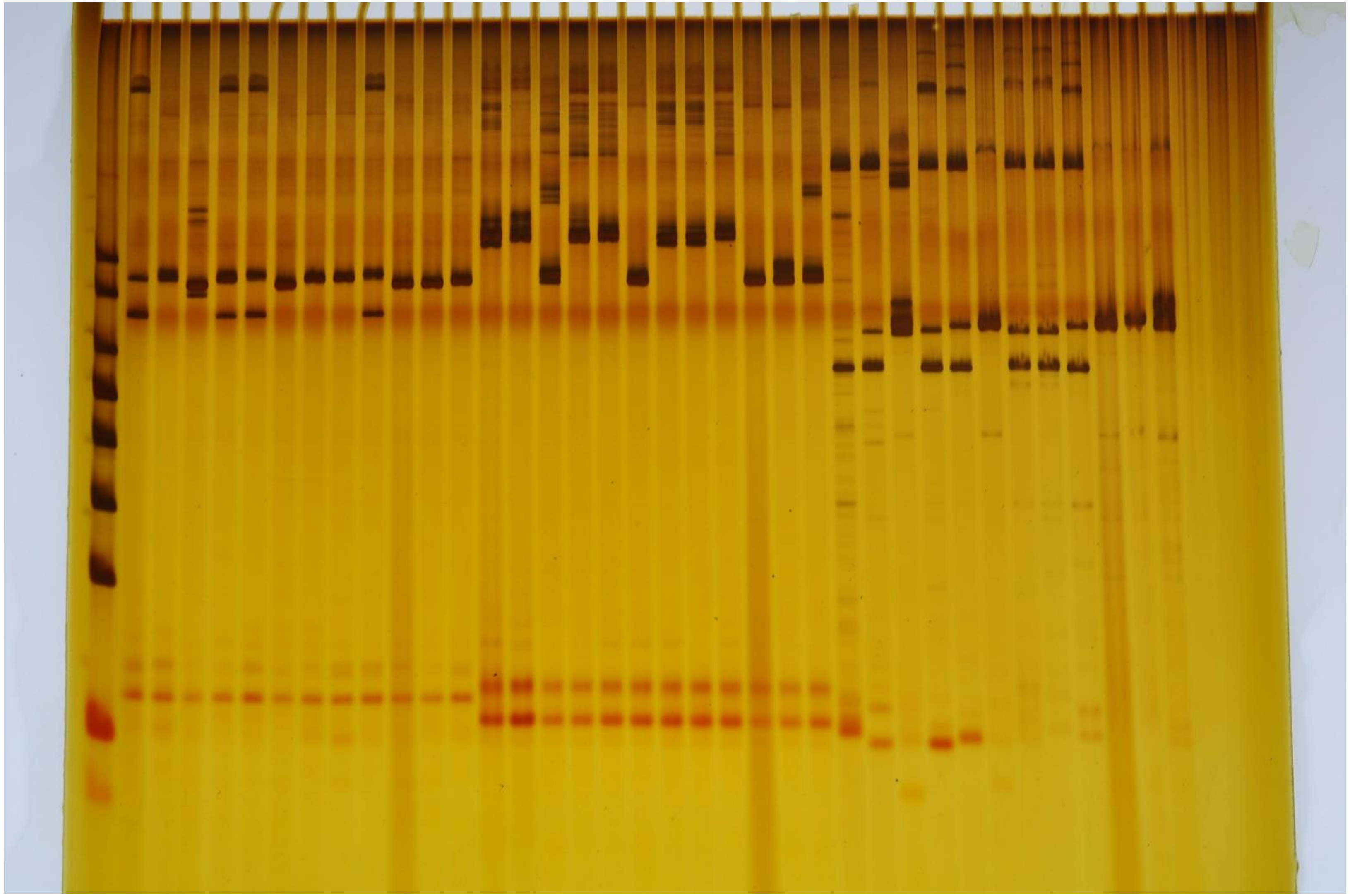

Chr8:   lane 2-lane 12
Chr15: lane 14-lane 24
Chr23: lane 26-lane 36

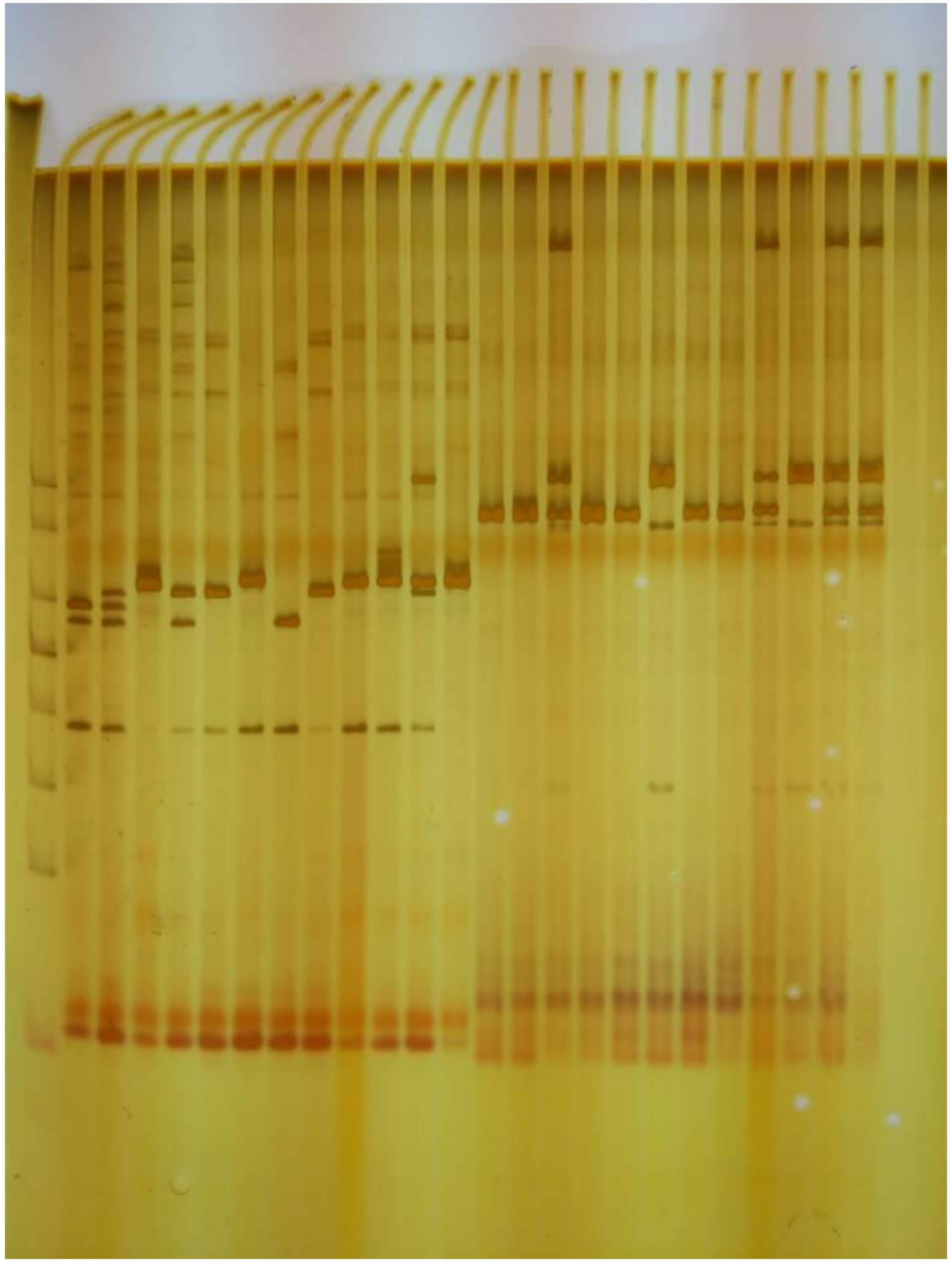

Chr12: lane 2-lane 12
Chr13: lane 14-lane 24

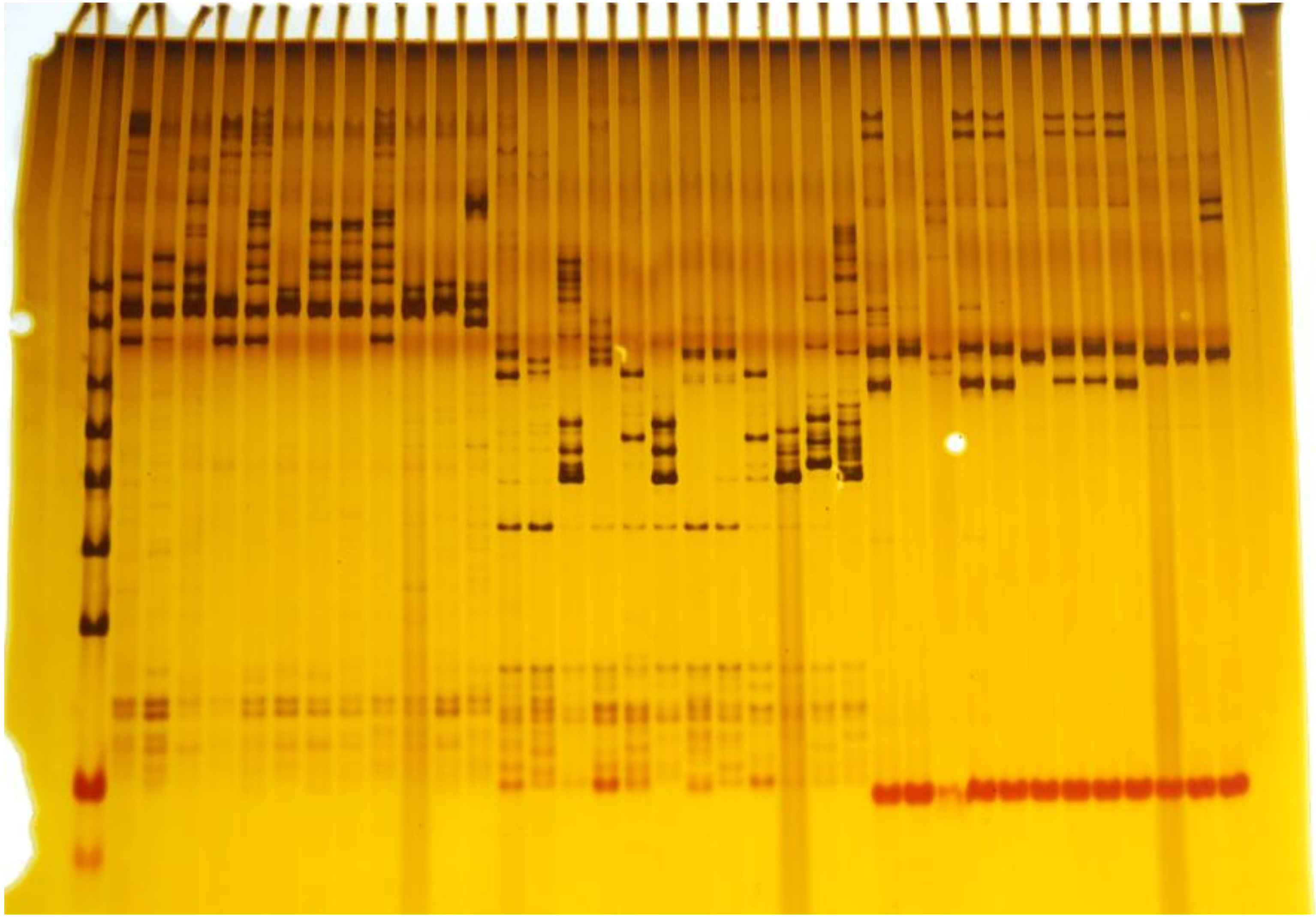

Chr27:   lane 2-lane 12
Chr100: lane 26-lane 36

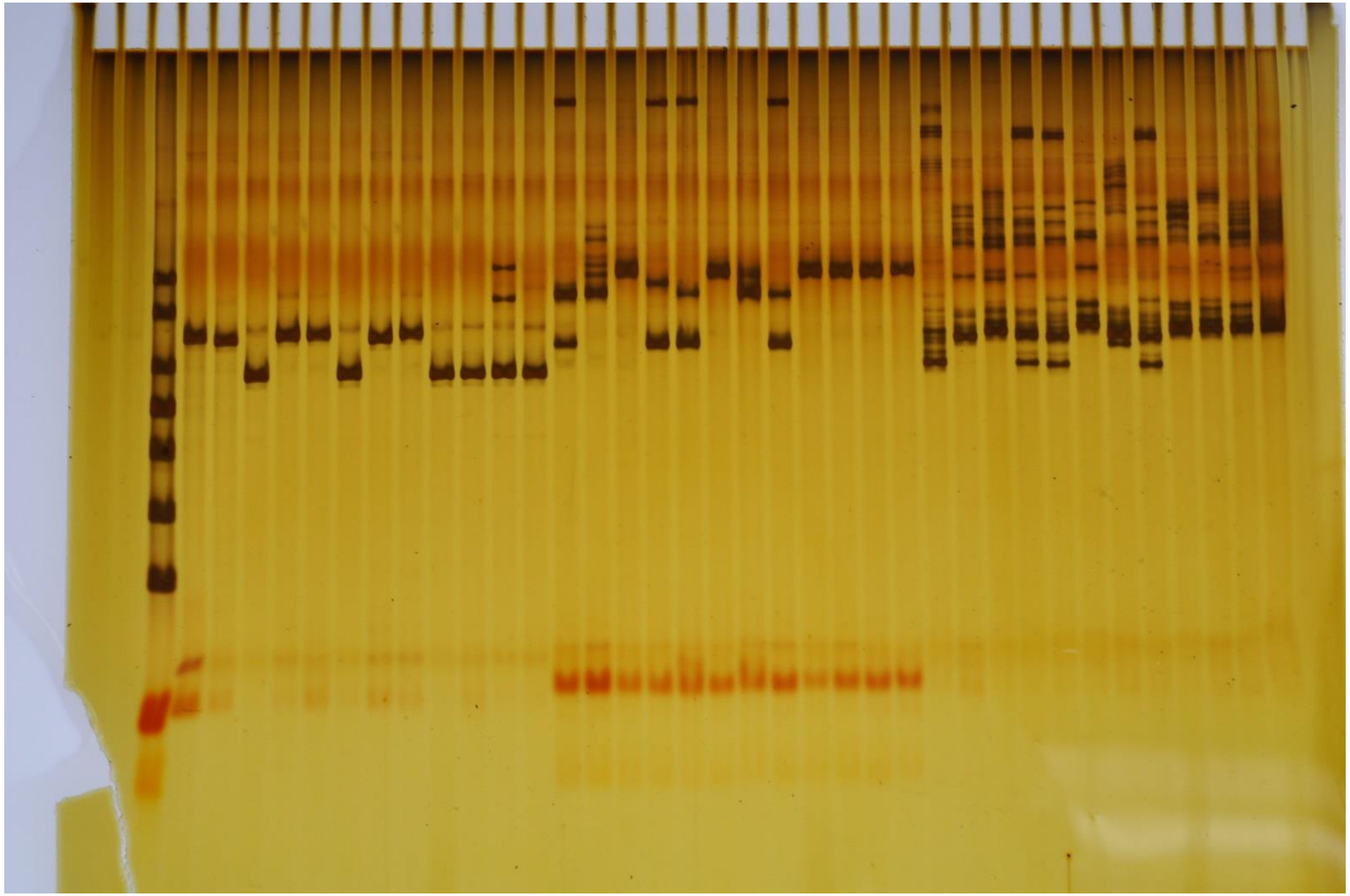

Chr39:   lane 2-lane 12
Chr44: lane 14-lane 24
Chr55: lane 26-lane 36

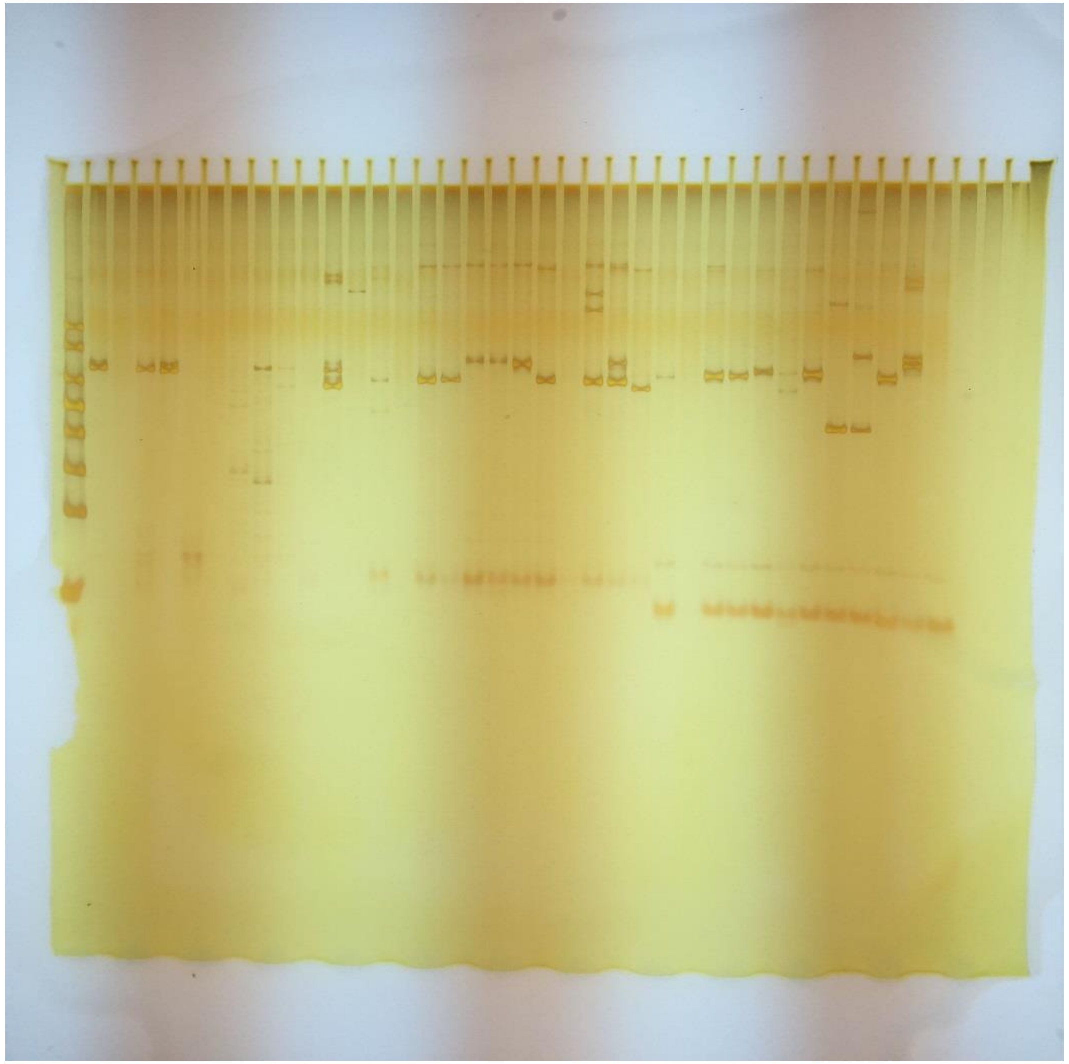

Chr52: lane 26-lane 36

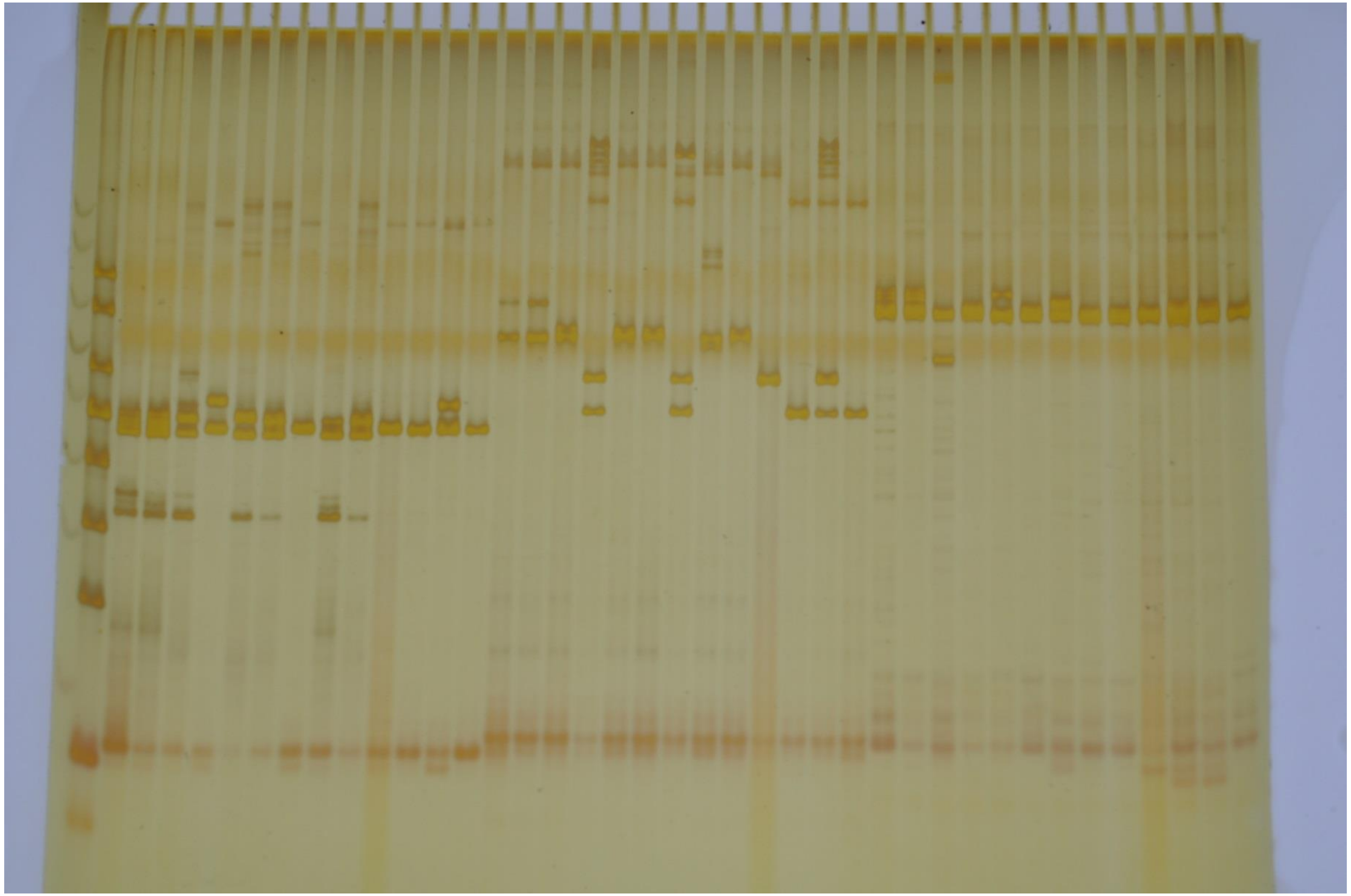

Chr93: lane 26-lane 36

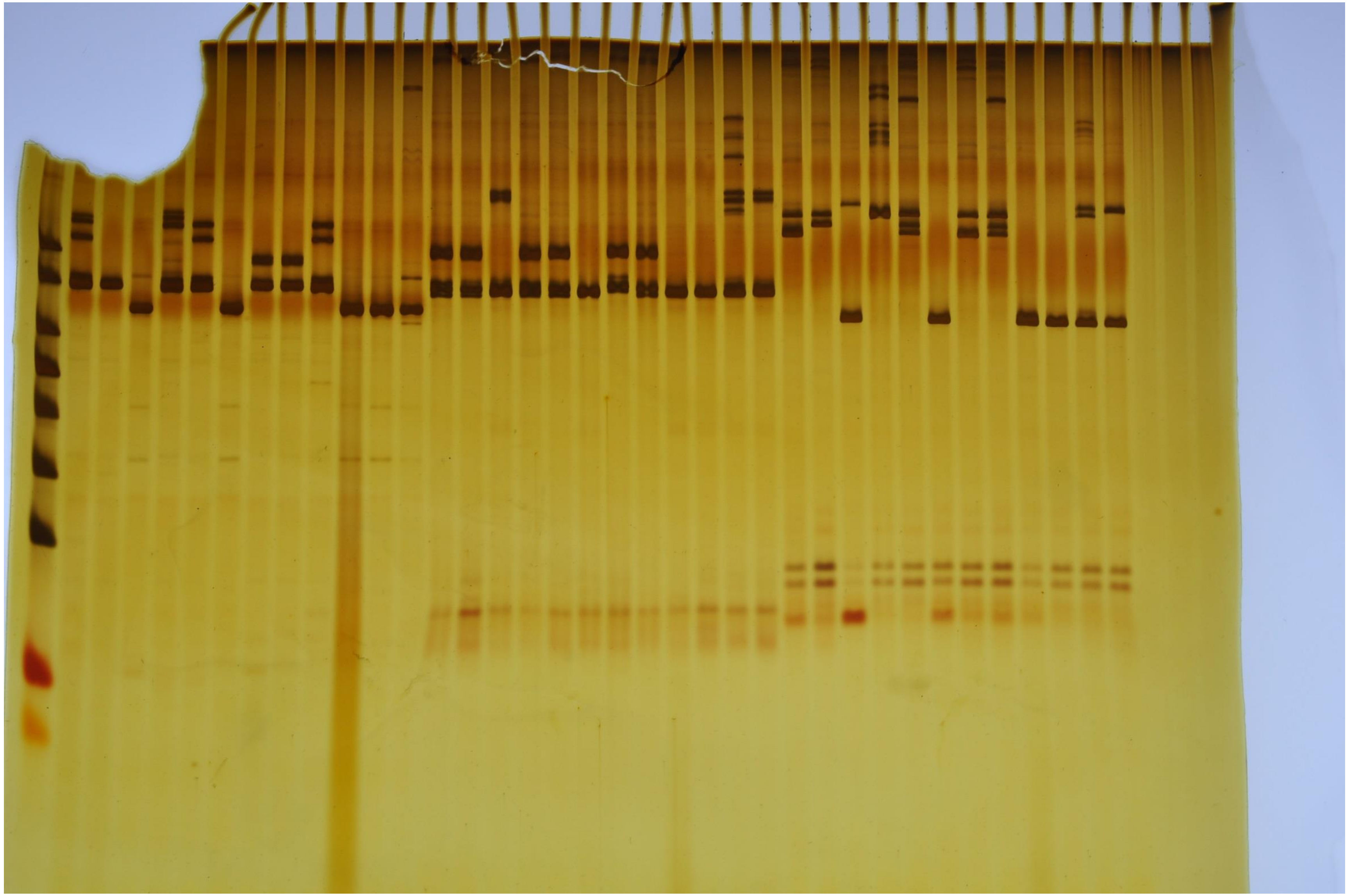

Chr18: lane 14-lane 24
